# Comparison of prenatal ultrasound with MRI in the evaluation and prediction of fetal orofacial clefts

**DOI:** 10.1186/s12880-022-00929-9

**Published:** 2022-12-05

**Authors:** Shuangshuang Gai, Lixiu Wang, Weizeng Zheng

**Affiliations:** 1 Department of Ultrasound, The Second Affiliated Hospital, Zhejiang University School of Medicine, Jiefang Rd No.88, Hangzhou, 310029 Zhejiang People’s Republic of China; 2 Department of Ultrasound, Women’s Hospital, Zhejiang University School of Medicine, Xueshi Rd No.1, Hangzhou, 310006 Zhejiang People’s Republic of China; 3 Department of Radiology, Women’s Hospital, Zhejiang University School of Medicine, Xueshi Rd No.1, Hangzhou, 310006 Zhejiang People’s Republic of China

**Keywords:** Prenatal diagnosis, Ultrasonography, Orofacial cleft, Magnetic resonance imaging, Predictive value of tests

## Abstract

**Background:**

Orofacial clefts (OFCs) are common craniofacial abnormalities. This study aimed to compare the diagnostic and predictive values of prenatal ultrasonography (US) and magnetic resonance imaging (MRI).

**Methods:**

We reviewed the newborn physical examinations or fetal autopsy data with OFCs. Between January 2013 and December 2018, the diagnoses resulting from prenatal US and MRI examination were compared retrospectively with the postpartum diagnoses. The diagnostic prediction of prenatal imaging was then determined.

**Results:**

334 infants were identified with OFCs by either newborn physical exam or stillborn autopsy. For detection of OFCs by US, the total accuracy (ACC), true positive rate (TPR), true negative rate (TNR), positive predictive value (PPV), and negative predictive value (NPV) were 99.9% (111,178/110,286), 81.9% (230/281), 99.9% (109,948/110,005), 80.1% (230/287), and 99.9% (109,948/109,999), respectively. For MRI, the ACC, TPR, TNR, PPV, and NPV were 99.8% (4,125/4,132), 89.8% (44/49), 99.9% (4,081/4,083), 95.7% (44/46), and 99.9% (4,081/4,086), respectively. When we compared the predictive values between prenatal US and MRI, there were significant differences in the PPV of OFCs (P < 0.05), NPV of OFCs (P < 0.05), TPR of CLO (P < 0.001), PPV of CLP (P < 0.05), and TPR of CPO (P < 0.05).

**Conclusion:**

Our results suggest that prenatal US could be effective for diagnosing and ruling out fetal OFCs. Diagnostic confidence is significantly improved when fetal MRI is used to assess fetal OFCs as an adjunct to US examination.

## Background

Fetal orofacial clefts (OFCs) are the most common congenital craniofacial anomaly, which impacts negatively on the life of the individual, and these defects occur in approximately 1.7 per 1000 live-born babies [1]. In current routine clinical practice, prenatal diagnosis of fetal OFCs is performed by ultrasound (US) and is reasonably accurate in most cases. With advances in US technologies, it may become easier to accurately diagnose a fetal OFC [2–4]. In future decades, further improvements in the expertise of sonographers should also result in continued increases in detection accuracy and rates [5–7]. OFCs may involve the lip, the primary palate and secondary palate, or the soft palate, and may also involve structures around the oral cavity, which can extend into the facial structures resulting in oral, facial, and craniofacial deformity [8]. However, the isolated cleft palate is difficult to detect on US images, and diagnosis of a cleft lip with or without cleft palate by US is not sufficiently accurate in primary care settings [7, 9, 10].

Fetal imaging is still a burgeoning topic. Magnetic resonance imaging (MRI) is an effective supplement to US and represents a valuable technique for diagnosing fetal facial deformities as MRI has the characteristics of a strong tissue contrast, multi-planar ability, good resolution, and is less affected by human factors [11, 12]. In-utero MRI can provide additional information regarding your baby’s diagnosis when the congenital malformation is detected on fetal ultrasound, and the value of MRI is potentially for isolated cleft palates as well which can be missed on ultrasound [13, 14]. In addition, the previous results demonstrated that the classification and degree of involvement of the cleft palate can be determined by fetal MRI [15]. More importantly, according to previous reports, there is currently insufficient evidence of the predictive probabilities of MRI alone in the diagnosis and classification of OFCs.

The present study aimed to collect fetal cases who were diagnosed with OFCs in antenatal or postnatal periods. The aim of this study was to report the predictive probabilities of prenatal US and MRI in the diagnosis of OFCs using larger sample sizes. Moreover, the objective of the present study was to compare the diagnostic values of prenatal US and MRI in the classification of OFCs.

## Methods

### Patients and setting

All clinical and imaging data were from patients enrolled at the Women’s Hospital, Zhejiang University School of Medicine, Hangzhou, China. This present study was approved by the Institutional Review Board, Women’s Hospital, Zhejiang University School of Medicine (Approval Number: IRB-20,200,059-R) while an informed consent was obtained from all individual participants and/or legal guardians. All methods were performed in accordance with the relevant guidelines and regulations of the National Health Commission of the People’s Republic of China. All data were coded without identifying details and were used for research purposes only.

In our hospital, from January 2013 to December 2018, the total number of births (including live births and stillbirths) was 110,286 (data from Hospital Digital Medical Record Systems (HDMRS)) (Shanghai Union Networks and Information CO., Ltd., Shanghai, China). We conducted keyword-based searches in HDMRS. We reviewed the newborn physical examinations or fetal autopsy data with OFCs (n = 334) from patients who delivered in our hospital over the same period, and then data were matched separately with intrauterine imaging findings from Picture Archiving and Communication Systems (Zhejiang Greenlander I.T. Co., Ltd., Hangzhou, China). Using postnatal physical examination or fetal autopsy as the “gold standard”, we analyzed the relationship between OFCs deformity and prenatal imaging. The true positive rate (TPR) (also called sensitivity), true negative rate (TNR) (also called specificity), positive predictive value (PPV), and negative predictive value (NPV) of OFCs were calculated separately and further compared.

### Prenatal imaging protocol

Although repeat scans were allowed, only the results and gestational ages of the first scans are reported in this article. In the present study, the arrangement of alveolar bone almost played a decisive role in the diagnosis and classification of OFCs for transabdominal ultrasound. In our cohort study, the diagnosis of cleft lip was confirmed or excluded by two-dimensional (2D) ultrasound, and then the hard palate was visualized in the 2D axial plane, after which 3D surface acquisition was performed with 3D ultrasound capability (Voluson 730 Pro, Voluson E8 Expert, Voluso E10 Expert; GE Medical Systems).

All prenatal MRI images were obtained using a 1.5-T unit (GE Signal HDxt) and an eight-element phased array body coil. The mothers were placed in a supine or left oblique position without sedation.

### Statistical analysis

Data were assessed using the software package SPSS version 21.0 (IBM SPSS Statistics for Windows, Armonk, NY, USA). The predictive probabilities were assessed by the TPR, TNR, PPV, and NPV. The analyses included student t-tests for continuous variables, Chi-square test for categorical variables. All statistical tests were two-sided, and *P*-values ≤ 0.05 were considered indicative of a statistically-significant difference.

## Results

### Patient characteristics

**Fig. 1:**
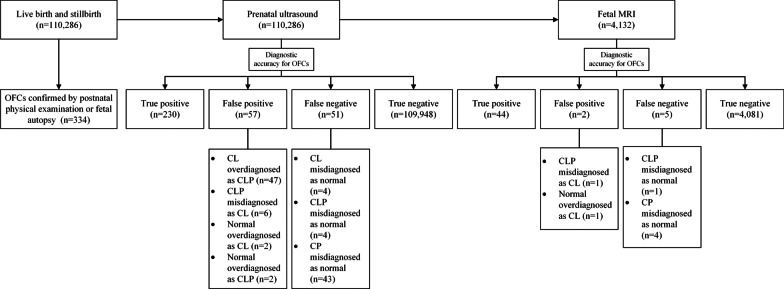
The flowchart shows the algorithm of statistical measures of OFCs. OFCs orofacial clefts

During the period analyzed, 334 babies were identified with OFCs by either newborn physical exam or stillborn autopsy (Fig. 1). The total incidence rate of OFCs was 3.03 per 1,000 births (334/110,286). Among these 334 babies, there were 73 cases of cleft lip only (CLO) (21.86%), 215 cases of cleft lip with cleft palate (CLP) (64.37%) and 46 cases of cleft palate only (CPO) (13.81%). Among 334 babies, 36 cases had undergone genetic testing and 20 cases were considered abnormal (Table 1). Eventually, 86 mothers (88 fetuses, two sets of twins) decided to continue with the pregnancy and four mothers with multiple pregnancies chose selective fetus reduction, while 246 mothers chose to terminate the pregnancy.

**Table 1:** Merging other malformations of fetal OFCs

n	Merging other malformations
2	Triplets
21	Twins
5	Trisomy 18
4	Trisomy 21
2	Trisomy 13
2	Microdeletion syndrome
7	Other chromosome abnormalities
71	Accompanied by syndrome and other malformations, such as Ichthyosis (n = 1), Pierre-Robin’s sequence (n = 1), Tetralogy of Fallot (n = 2), velocardiofacial syndrome (n = 3), holoprosencephaly (n = 3), Dandy-Walker malformation (n = 2), diaphragmatic hernia (n = 2), abnormal development of cloacal cavity (n = 3), congenital heart disease (n = 22), limb and spine deformity (n = 14), facial dysplasia (n = 8), and so on (n = 10)

*OFCs* orofacial clefts

In China, prenatal US screening throughout pregnancy was made universally accessible by legislation and health insurance coverage, and includes prenatal screening, prenatal diagnosis, and growth measurements. Throughout prenatal screening in our hospital, we believe that almost all pregnant women (110,286 births) have had at least one prenatal US examination. In our current study, prenatal US diagnosed 287 fetuses with OFCs (30 cases of CLO, 254 cases of CLP, three cases of CPO), which included four false-positive cases who were misdiagnosed as with CLO (two cases) and CLP (two cases), and failed to detect 51 fetal OFCs (four cases of CLO, four cases of CLP, 43 cases of CPO) (Table 2). The total accuracy (ACC) of prenatal US for OFCs was 99.90% (110,178/110,286). Of the 338 births analyzed, TPR, TNR, PPV, and NPV of total OFCs were 81.85% (230/281), 99.95% (109,948/110,005), 80.14% (230/287), and 99.95% (109,948/109,999), and CLO, CLP, CPO were shown the same values as above in Table 3. The mean gestational age at US diagnosis was 25.05 ± 3.95 weeks (range 15–39).

**Table 2:** Comparison of total prenatal imaging and follow-up medical records

Prenatal diagnosis		Postnatal diagnosis
Ultrasound detection	MRI detection	
CLO (n = 2)	CLO (n = 1)	Normal
CLO (n = 22)	CLO (n = 11)	CLO
CLO (n = 6)	CLO (n = 0)	CLP
CLP (n = 2)	CLP (n = 0)	Normal
CLP (n = 47)	CLP (n = 1)	CLO
CLP (n = 205)	CLP (n = 30)	CLP
CPO (n = 3)	CPO (n = 3)	CPO
Normal (n = 4)	Normal (n = 0)	CLO
Normal (n = 4)	Normal (n = 1)	CLP
Normal (n = 43)	Normal (n = 4)	CPO
Normal (n = 109,948)	Normal (n = 4,081)	Normal
Total (n = 110,286)	Total (n = 4,132)	–

*OFCs* orofacial clefts, *CLO* cleft lip only, *CLP* cleft lip with cleft palate, *CPO* cleft palate only, Normal, fetal lip and palate are normal

**Table 3:** Rates of predictive characteristics of OFCs by prenatal US and fetal MRI

Characteristics	CLO			CLP			CPO			Total		
	US	MRI	P value	US	MRI	P value	US	MRI	P value	US	MRI	P value
TPR (%)	22/73 (30.14)	11/12 (91.67)	< 0.001	205/215 (95.35)	30/31 (96.77)	0.916	3/46 (6.52)	3/7 (42.86)	0.029	230/281 (81.85)	44/49 (89.80)	0.246
TNR (%)	110,205/110,213 (99.99)	4,119/4,120 (99.98)	0.753	110,022/110,071 (99.96)	4,100/4,101 (99.98)	0.822	110,240/110,240 (100)	4,125/4,125 (100)	1.000	109,948/110,005 (99.95)	4,081/4,083 (99.95)	0.785
PPV (%)	22/30 (73.33)	11/12 (91.67)	0.372	205/254 (80.71)	30/31 (96.77)	0.049	3/3 (100)	3/3 (100)	1.000	230/287 (80.14)	44/46 (95.65)	0.019
NPV (%)	110,205/110,256 (99.95)	4,119/4,120 (99.98)	0.781	110,022/110,032 (99.99)	4,100/4,101 (99.98)	0.865	110,240/110,283 (99.96)	4,125/4,129 (99.90)	0.158	109,948/109,999 (99.95)	4,081/4,086 (99.88)	0.031

*OFCs* orofacial clefts, *CLO* cleft lip only, *CLP* cleft lip with cleft palate, *CPO* cleft palate only, *TPR* true positive rate, *TNR* true negative rate, *PPV* positive predictive value, *NPV* negative predictive value

In total fetal MRI examinations (n = 14,611) during the same period, 4132 babies who had undergone fetal MRI due to suspected fetal anomalies were born in our hospital. In 4132 babies, 51 babies were related to OFCs. MRI diagnosed 46 cases of OFCs (12 cases of CLO, 31 cases of CLP, three cases of CPO) (Table 2). Compared with birth medical records, MRI failed to detect OFCs in five cases (one CLP, four CPO). TPR, TNR, PPV, and NPV of total OFCs were 89.80% (44/49), 99.95% (4081/4083), 95.65% (44/46), and 99.88% (4,081/4,086) (Table 3). For all MRI, the total ACC for OFCs was 99.83% (4125/4132). Of 51 fetuses, the mean gestational age at MRI diagnosis was 27.67 ± 3.74 weeks (range 23–37).

### Comparing US and MRI diagnosis

When comparing the US and MRI diagnosis in the overall cohort, the total ACC of identification of OFCs did not differ significantly (*P* = 0.15). The comparison between the predictions made by prenatal US and MRI is shown in Table 3, and US examination allowed good prediction of all OFCs. Based on these results, we firmly believe that US examination has been regarded as a standard modality in the evaluation of fetal OFCs in the entire population.

However, in our assessment of US examination, 47 cases of CLO were misdiagnosed as CLP and six cases of CLP were diagnosed as CLO, so this caused the lower TPR (30.14%) of CLO, PPV (73.33%) of CLO, and PPV (80.71%) of CLP. Additionally, TPR, TNR, PPV, and NPV of the cleft lip with or without cleft palate was 97.22% (280/288), 99.99% (109,994/109,998), 98.59% (280/284), and 99.99% (109,994/110,002), respectively. Hence, prenatal US cannot clearly distinguish between cleft lip alone or combined with cleft palate. Simultaneously, there were significant differences (*P* < 0.05) in the PPV and NPV of total OFCs, when we compared the predictive probabilities between prenatal US and MRI. Furthermore, there were significant differences in the TPR of CLO (*P* < 0.001), PPV of CLP (*P* < 0.05), and TPR of CPO (*P* < 0.05). In addition, 43 cases of CPO were missed by prenatal US and TPR of CPO was only 6.52% (3/46). Moreover, TPR of CPO was 42.86% (3/7) for MRI. Based on these results, we can confirm that MRI is an effective predictive method to supplement US for fetal OFCs, particularly in distinguishing cleft lip with or without cleft palate.

## Discussion

The accuracy and technique of prenatal US in diagnosing OFCs have been the subject of several previous studies [16, 17]. Prenatal US is an integral part of prenatal care. Our findings identify that MRI is a useful adjunct to prenatal US in the diagnosis of OFCs and represents a valuable technique for fetal diagnosis of different classifications of OFCs. Furthermore, we present the predicted values of prenatal US and MRI from a large sample of patients screened at our center.

With more families presenting in the prenatal period, it is critical for prenatal diagnosticians and plastic surgeons to understand the techniques in use today for prenatal cleft diagnosis, as well as their associated limitations [18]. At present, prenatal diagnostic US is widely used for the detection of OFCs. Prenatal detection rates revised by Maarse et al. [19] ranged from 9 to 100% for cleft lip with or without cleft palate, 0–22% for CPO and 0–73% for all types of cleft. Lai et al. [20] tried to evaluate the sensitivity and specificity of US for detecting cleft palate in high-risk fetuses, and pooled sensitivity was 87% (95% CI 71–95%) and pooled specificity was 98% (95% CI 90–100%). However, there have been few reports about TPR, TNR, PPV, or NPV of OFCs based on a large number of birth records, and a lack of detailed explanation about the proportion of actual positives and negatives that are correctly identified. In our present study, for the prediction by US examination, TPR, TNR, PPV, and NPV of total OFCs were very satisfactory (81.85, 99.95, 80.14, and 99.95%, respectively), and the sensitivity and specificity were basically consistent with previous reports [21, 22]. However, for US examination, the TPR of CLO was 30.14%, and the PPV of CLO and CLP was 73.33% and 80.71%, respectively. The cause of the above values was that 47 cases of CLO were misdiagnosed as CLP and six cases of CLP were diagnosed as CLO. We found that prenatal US did not provide a better identification rate for patients with cleft lip combined with or without cleft palate. This demonstrates the need for us to identify effective complementary technology with which to compensate for the limitations of US in the diagnosis of OFCs.

According to existing literature [12, 23], the relevance of diagnostic accuracy in assessing MRI is self-evident, especially to compensate for the limitation of US in the diagnosis of cleft palate. Furthermore, MRI demonstrated the classification and degree of involvement of the cleft palate [15]. Although Descamps et al. [24] and Laifer-Narin [25] also reported the PPV, NPV, sensitivity, and specificity of MRI for the involvement of cleft palate, we have not yet found one report on four predictions of OFCs using a large sample of birth records and fetuses of various gestational ages. More importantly, we compared the values of US and MRI in the diagnosis of OFCs during the same period. For MRI, the TPR, TNR, PPV, and NPV of all OFCs were 89.80, 99.95, 95.65, and 99.88%, respectively. In addition to CPO, MRI was more effective in correctly identifying fetuses with or without OFCs. Concomitantly, there was a significant difference (*P* < 0.05) between the prenatal US and MRI for the PPV of total OFCs, NPV of total OFCs, TPR of CLO, PPV of CLP and TPR of CPO, when we compared the predictive ability between the two groups. We found that the sonographers may have been more likely to overdiagnose CLO, and MRI examinations in a high-risk setting provided more opportunity to detect additional cleft palates. Moreover, when OFCs are detected on screening, MRI is helpful in allowing the parents and the prenatal counseling team to obtain accurate information, whether or not the hard palate is cleft. In our present study, our results show that MRI could improve the processing capacity for prenatal diagnosis and enable us to accurately predict the occurrence of OFCs to guide clinical management.

The major limitation of our study is that it is a single-center retrospective study. According to previous data, there are fewer misdiagnosed cases with MRI, which has a higher diagnostic predictive value for cleft lip and palate, so the results are inevitably somewhat biased. At the same time, CPO is a worldwide problem, and the search for ways to improve its diagnostic ability makes our work worthy of attention [3, 10, 26]. We will continue to supplement our data to improve the results.

## Conclusion

Our results suggest that, for prenatal US, the high specificity and sensitivity are effective in diagnosing and ruling out OFCs by analyzing large amounts of information. Diagnostic confidence is also improved when MRI is used to assess prenatal fetal OFCs as an adjunct to US. Enhanced technology and increased confidence result in changes in counseling and clinical management for the obstetrician and radiologist.

## Data Availability

The datasets used and/or analysed during the current study are available from the corresponding author on reasonable request.

## Abbreviations

OFCs: Orofacial clefts
US: Ultrasonography
MRI: Magnetic resonance imaging
ACC: Accuracy
TPR: True positive rate
TNR: True negative rate
PPV: Positive predictive value
NPV: Negative predictive value
HDMRS: Hospital digital medical record systems
2D: Two-dimensional
CLO: Cleft lip only
CLP: Cleft lip with cleft palate
CPO: Cleft palate only
